# Re-evaluation of direct hemoperfusion with polymyxin-B immobilized fiber for severe sepsis and septic shock

**DOI:** 10.1186/cc9540

**Published:** 2011-03-11

**Authors:** SM Matsuo, TI Ikeda, KI Ikeda

**Affiliations:** 1 Tokyo Medical University, Hachioji Medical Center, Tokyo, Japan

## Introduction

The equivalency of continuous venovenous hemofiltration and intermittent hemodialysis (2B) was described as a key recommendation of the Surviving Sepsis Campaign guidelines in 2008. However, there are some discrepancies associated with the evaluation of blood purification in severe sepsis and septic shock in Japan. Direct hemoperfusion with polymyxin-B immobilized fiber (PMX-DHP), developed and currently in use in Japan, has not yet been evaluated abroad. We performed a retrospective study to re-evaluate PMX-DHP for severe sepsis or septic shock patients in our ICU.

## Methods

We enrolled 302 patients (survival (S) group: 201, nonsurvival (NS) group: 101) in whom PMX-DHP had been performed for severe sepsis and septic shock from 1994 to 2010. These patients were allocated into two groups: those who survived for at least 28 days after the start of PMX-DHP therapy (S group: 201 patients) and those who did not (NS group: 101 patients). Background factors (age, gender, APACHE II scores, sepsis-related organ failure assessment score, Goris multiple organ failure (MOF) score), hemodynamics (blood pressure, PaO_2_/FiO_2 _ratio, catecholamine requirement), inflammatory mediators (IL-6, IL-8, IL-1ra), endothelial-related markers (PAI-1, ELAM-1) and procalcitonin levels were examined in each group.

## Results

On background factors, only the Goris MOF score showed a statistically significant difference among the groups. Blood pressure and the PaO_2_/FIO_2 _ratio both improved markedly immediately after PMX-DHP. Also, the average required amount of catecholamine decreased after PMX-DHP. IL-6 and IL-1ra levels decreased immediately after PMX-DHP in both groups, but these values before PMX-DHP did not show any statistically significant difference between the groups. PAI-1 levels showed a significant decrease after PMX-DHP in both groups.

## Conclusions

We confirmed an improvement in pulmonary oxygenation and hemodynamic parameters using PMX-DHP for severe sepsis and septic shock patients. The levels of various inflammatory mediators decreased using PMX-DHP, but we did not find any correlation between these changes and outcome.

